# The unbroken chain of female genital mutilation: a qualitative assessment of high school girls’ perspectives

**DOI:** 10.1186/s12905-023-02843-w

**Published:** 2024-01-03

**Authors:** Lina Hemmeda, Lena Anwer, Marwa Abbas, Lina Elfaki, Maram Omer, Maab Khalid, Mushrega Hassan, Mihrab Mostafa, Lina Hamza, Maab Mahmoud, Maram Mohamed Osman, Mozan Mohamed, Lamees Bakheet, Alaa T. Omer

**Affiliations:** Faculty of Medicine, University of Khartoum, P. O. Box 11111, Khartoum, Khartoum Sudan

**Keywords:** Female genital mutilation, Perception, Traditions, Women’s rights

## Abstract

**Background:**

Female Genital Mutilation (FGM) is defined as any procedure that involves damage to the female external genitalia. This practice is majorly prevalent in Sudan, as it is estimated that over 12 million Sudanese women are circumcised. This study uncovers rural females’ knowledge and insights about FGM domestically.

**Methods:**

A qualitative, deductive study with thematic analysis was conducted. A total of 42 female high school students were recruited and divided into five focus groups, each of which included girls from four different high school classes in the main school of the study area. A topic guide was prepared and used to lead the focus groups. Thematic analysis was used, and the study data had been categorized into four themes: knowledge, procedure and performance, experience, and practice. The condensed meaning units of each theme were identified, then classified to formulate sub-themes.

**Results:**

All the participants indicated that FGM is a traditional practice in the village. The vast majority have heard about it from family members, mainly mothers and grandmothers. Regarding the procedure, all the participants agreed that midwives perform FGM, but most of them don’t know what exactly is being removed. According to all participants, mothers and grandmothers are the decision-makers for FGM. The majority of the participants stated that they do not discriminate between the circumcised and uncircumcised women and most of them agreed that circumcision has negative side effects. They have mentioned pain, difficult urination, and walking as early side effects, while psychological impacts and labor obstruction as late ones. Generally, the majority of the participants agreed that circumcision is not beneficial and should stop.

**Conclusion:**

Knowledge regarding the dangers of FGM among high school girls is better than expected given the high prevalence of the practice. Generally, the process is well understood, the performers are known, the experience is universal, and the side effects are acknowledged. Nevertheless, a majority still showed an intent to circumcise their daughters in the future.

## Introduction

The World Health Organization (WHO) and United Nations Children’s Fund (UNICEF) jointly classified Female Genital Mutilation (FGM) as any procedure involving the partial or complete removal of the external genitalia, namely the clitoris, the labia minora and the labia majora, or any harm to the female genital organ [1]. The terms “female circumcision,” “female traditional surgery,” “female cutting,” and “excision” are also used to describe this custom [1]. Additionally, many other global health and developmental organizations, such as the department for international development (DFID) and US Agency for International Development (USAID) classified it as common and harmful practice [2, 3]. In Africa, the Middle East, and Asia, FGM is a prevalent ritual, it carries a significant danger for long-term issues with one’s bodily and sexual well-being [4]. The WHO lists sociocultural, hygienic and aesthetic, spiritual and religious, and ultimately economic considerations as the cultural justifications for FGM practice. For instance, many cultures hold the view that uncircumcised women may purposefully blind anyone who witnesses them or even kill their husband. Additionally, ladies who have not been circumcised typically have little to no chance of getting married, which could introduce a financial burden on the family [5]. There are four main types of FGM, ranging in severity from type I to IV. In type I, the clitoris is removed, either partially or totally (clitoridectomy) but sometimes only the prepuce is removed. In type II, the labia minora is excised in addition to the clitoris, and the labia majora may be cut as well. Type III is considered as the most severe form of FGM, and is referred to as infibulation, it’s characterized by the sealing of the vulva completely leaving only a small hole for passing both urine and menstrual blood through the cutting and apposition of the labia majora and/or minora, with or without excision of the clitoris. Type IV encompasses all other forms of harmful genital manipulations [3]. Type III is the most severe form with a 10% global prevalence [2]. In terms of geographical distribution, type I is commonly practiced in Mali, Nigeria, Burkina Faso, and Senegal, while type II is mainly practiced in Sudan and Burkina Faso. Type III is considered 15% of FGM and is frequently practiced in Djibouti, Egypt, the Gambia, Mali, Eritrea, Somalia, and Sudan [6].

With the extent of FGM, some long-term obstetrical, genitourinary, and psychosexual health issues occur more frequently and are more severe. Genitourinary issues include keloids, dysmenorrhea, hematometra, hematocolpos, urinary tract infections, dribbling, weak urine flow, and protracted micturition. Increased risks of cesarean sections, episiotomies, perineal rips, postpartum hemorrhage, and stillbirths are among the obstetrical issues [7, 8]. The signs of anxiety, depression, dyspareunia, a lack of sexual desire, and decreased sexual satisfaction are all examples of psychosexual health issues [9]. For all the aforementioned, the International Federation of Gynaecology and Obstetrics (FIGO) condemns any attempt to medicalize the process or facilitate its performance at health institutions or by medical providers under whatever circumstances [10].

Girls and women with FGM-related health issues require specialized medical care, including clitoral reconstruction and deinfibulation, along with enhancing sexual function and self-esteem in women and girls who underwent any sort of procedure involving clitoris excision [11]. Moreover, the body image, self-confidence, and ultimately the sexual function of women and girls who have undergone FGM are thought to be improved with the help of psychosexual counseling, which is considered to be both risk-free and successful. Therefore, psychosexual enhancement of sexual function is advised by the WHO guidelines on the management of FGM-related health problems rather than clitoral repair [12].

An estimated 100 to 140 million women worldwide are living with the consequences of FGM [13]. In Africa, an estimated 92 million girls 10 years of age and older have undergone FGM, with an average of four girls each minute being mutilated [14]. Despite being a dangerous and negative practice, there are still many deeply rooted beliefs and cultures in most of Sudan’s rural areas that glorify FGM as a necessity for every female, the most recent data show that 86.6% of women and girls in Sudan between the ages of 14 and 49 still experience FGM. It is estimated that over 12 million women and girls have experienced FGM [15].

This study sheds light on rural high school girls’ perspectives about FGM in terms of knowledge and experience, with the ultimate aim of preventing Female Genital Mutilation (FGM) and to promote sex equity as well as human dignity. We thought that a qualitative assessment of high school’s girls perspectives and attitudes would be such a helpful tool to, firstly, understand the evolvement of these perceptions and practices in the context of their cultural background; besides, qualitative research can dig in and uncover the psychological impact of this culture in both girls who have personally experienced FGM and those who have been under social and peer pressure pushing them toward embracing this harmful practice. By this, we are trying to provide a more culturally sensitive insight about FGM for the community leaders to address this issue. Additionally, it serves to enrich Sudan deficit literature with more information about rural resident’s perspectives.

## Materials and methods

### Study design and setting

This is a qualitative-based deductive thematic analysis carried out in December 2022 at Wad-Elhabashi Secondary School for Girls in Wad-Elhabashi Village, Al-Matama Locality, River Nile State, Sudan. The study accompanied a rural residency program that targeted the study area. Wad-Elhabashi village has been chosen by the community medicine department of the university of Khartoum to be the target of a health education program that serves as a social accountability tool to engage the fifth year student with the rural community through health education and health promotion activities. As well, it trains fifth year students about various aspects of the rural healthcare system and culture. The village is situated on the western bank of the Nile, about 120 km north of Khartoum, the capital of Sudan, combining rural and urban lifestyles. Roughly ten thousand people live there, most of whom are educated, and most of them are farmers. The community frequently engages in FGM, as observed by the health facility, which is consistent with Sudan’s high FGM prevalence [15].

### Sampling

Purposive sampling was used to recruit students from different classes and divide them randomly into five focus group discussions (FGDs); based on a profile comprising a set of selection criteria, including all those attending Wad-Elhabashi Village High School who were willing to voluntarily participate in the study, were comfortable talking about the potentially sensitive topic of FGM, and are able to provide informed consent to participate in the focus groups. A purposive sample was used as per the recommendations of Moser et al. to ensure that potential informants could facilitate the answer to our research question [16].

### Data collection procedure

Before the data collection, a training session was held for the data collectors, during which an expert researcher produced a simulated session to provide the collectors with real life experience and to ensure a good readiness for qualitative research methods. The expert showed multiple techniques and focused in interviews and focus groups, and emphasized participant observation in light of this sensitive topic. Then, the research objectives and questions were thoroughly discussed until each data collector was fully understanding the overall purpose of the topic and the qualitative method. In the light of the above steps, FGD guide questions and instructions were discussed and described. The issue of cultural sensitivity has been discussed, and the data collectors were told and reminded to respect the local norms and beliefs while interviewing the participants; they were also told to expect any sort of apprehension and reminded to respect it with no further pressure. An effective note taking technique was recommended and implemented, where a companion data collector was dedicated exclusively to this mission. Each of the collectors was a fifth-year medical student.

On the day of the data collection, the selected participants were distributed into five FGDs, each facilitated by three researchers, a moderator who guided the discussion, a co-moderator who observed and managed the voice recordings, and a writer, who was responsible for taking notes. Due to a lack of proper facilities inside the school, the front yard was deemed the most environmentally suited location for FGDs. We did, however, ensure that all other students remained confined in their classrooms with a teacher monitoring them, and that the rest of the teachers were gathered in the school’s principle office, setting for an entirely private environment with no by-passers throughout the sessions. Groups were distanced to provide a more relaxed and accommodating feel.

Participants were informed of the purpose of the gathering and informed of their right to decline or to leave the discussions if they felt uncomfortable to continue A discussion guide was prepared by the authors after reviewing the literature [17]. It was then divided into opening and introductory questions, which aimed at breaking the ice and indulging the participants in the discussion environment, transitional questions, which serve to prepare them for the study questions, and key questions to achieve the study purpose. A questionnaire with open questions was used, the key questions were semi-structured into questions about FGM knowledge and questions about the experience of FGM; Table 1 demonstrates the questions corresponding to each theme. Furthermore, the sessions took 40–50 min for each and were conducted in Arabic, recorded and transcribed, then translated into English.

**Table 1:** The questions corresponding to each theme and subthemes

Theme	Sub themes	Questions
**FGM knowledge**	Concept of FGM	Have you heard about FGM?
	Sources of the information	From where did you hear about it?
	Procedure of FGM	Do you know how exactly FGM is done?
**People involved in** **FGM**	Executor	Who usually performs FGM
	Decision maker	Who usually makes the decision of FGM
**Experience**	Negative effects	Does FGM have any bad effects? (Whether immediate or late)
	Positive effects	Does FGM have any beneficial effects? (Whether immediate or late)
Attitude	Discrimination	Is there a difference between being circumcised and not? (Culturally, religiously, etc.)
	Action towards FGM	Do you think this practice should be continued or should be stopped?

### Data Analysis

**Fig. 1:**
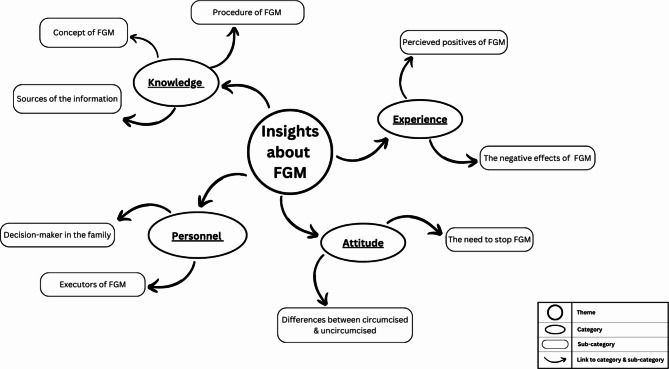
Visual overview of the study themes

The data had been coded and analyzed manually through thematic analysis, following the six steps reported by Braun and Clarke [16]. First, the transcripts were thoroughly reviewed by four authors independently. Second, collectively, they highlighted condensed meaning units from the data and generated codes, quotes relevant to each code were extracted and tabulated to illustrate the results. Third, codes were linked into sub-categories. Fourth, each similar group of subcategories was collected under a category, or theme. Fifth, themes were reviewed 10 and finalized by a different author. Sixth, two authors defined and named each theme. Figure 1**displays the final study themes.** Data saturation was reached when we detected that no new relevant information was emerging [18]. Therefore, we limited the sample size to 42 participants.

## Results: insights about FGM

A total number of 42 participants were involved in this study. The mean age of the participants was 15 ± 1.09 years old but the vast majority (63.6%) aged between 13 and 15. Regarding parental education and due to the statements of the participants, 43.2% of the mothers have had secondary education; 22.7% of the fathers have had only primary education while most of the fathers’ educational statuses were unknown to their daughters who we have interviewed. The socio-demographic characteristics of the participants are shown in Table 2.

**Table 2:** The details of the socio-demographic characteristics of the participants

Variables	Categories	*N*	%
Age (years)	(13–15)	**28**	**63.6**
	(16–18)	**16**	**36.4**
Mother’s education level	Illiterate	**3**	**6.8**
	Informal (khalwa)	**2**	**4.5**
	Primary	**16**	**36.4**
	Secondary	**19**	**43.2**
	University	**4**	**9.1**
Father’s education level	Illiterate	**2**	**4.5**
	Informal (khalwa)	**0**	**0**
	Primary	**11**	**25**
	Secondary	**10**	**22.7**
	University	**3**	**6.8**
	Unknown	**18**	**40.9**

### Category 1: FGM knowledge among high school girls

The participants expressed their understanding of the various aspects that characterize FGM, and the findings are summarized in the subsequent sub-categories: the concept of FGM, the sources of the information, and the procedure of FGM.

### The Concept of FGM

Regarding the concept of FGM, all participants who were asked about the idea of FGM said that it is a customary practice in the village. Despite being all circumcised, the girls referred either to their neighbors or the village as a whole while discussing the procedure performance.

*“Yes, our neighbors perform it*” (A participant from group 5).

“*They do it for marriage; it reduces the woman’s sexual desire*” (A participant from group 2).

### The sources of the information

The vast majority claimed to have learned about it from family members, typically their mothers or grandmothers, “*I heard my mother and aunts talk about it after a girl in our neighbor was operated, I asked my mother and she explained to me all about it and told me that I and every girl in the village have had to do it*”. However, very few stated to have learned about it from society as a whole, except one participant who said to have only learned about it from social media, as a prohibited activity.

“*I have heard about it from social media and it’s a forbidden thing*” (A participant from group 3).

### The Procedure of FGM

Most participants admitted to cutting and sewing something, but they were unsure of what it was. However, some of them claimed to cut and sew the clitoris and to practice type I “Sunni,” while the rest insisted on practicing type II “Pharaonic.” Interestingly, those who practiced Type I were more likely to have mothers with high school or college degrees. This correlation may indicate that educated families typically practice a relatively lighter form of FGM than the rest of the village.

“*Until now, I did not know what they did or what they cut*.” (A participant from Group 3).

“*I think they usually cut the clitoris—just a small piece of it. I think it is “Sunni*” (A participant from group 2).

“*They perform the “Pharaonic” type and cut a large piece of the genitalia*” (A participant from group 5).

### Category 2: FGM involved personnel

While discussing the process of FGM, participants mentioned various people who are potentially involved in the procedure; and categorized them into two sub-categories: executors of FGM and decision-makers in the family.

### Those who perform FGM

Participants who were in particular previously active in explaining the procedure of FGM were noted to mention that traditional birth attendants, midwives, or elderly women perform FGM, and when asked if doctors were involved, all agreed that they are not involved, with a few pointing out that they are not needed, while others did not know why. While the remainder provided additional reason, according to one participant, it is harmful; “The doctor does not perform it because it is harmful and alters God’s creation”. Another participant stated, “The midwives perform FGM, but doctors do not perform it because it is illegal and forbidden”. A third added, “Doctors perform circumcision for boys only, but midwives perform it for girls”. One participant stated that she would prefer doctors to perform it for her safety.

“*It is done by a midwife, but it is preferable to be done by a doctor*.” (A participant from group 5).

### The decision-makers in the family

According to the participants, the mother or grandmother always decides whether or not to perform FGM on their daughters. Some claimed that the decision is made solely by the mother, while others asserted that the decision is made by the grandmother. Generally, men or fathers are not involved in such decision-making.

“*Mothers, grandmothers, and aunts are more likely than fathers to decide to perform FGM on their daughters*.” (A participant from Group 4).

*“Our grandmother is the only one who makes these decisions in our family.”* (A participant from group 3).

One participant stated that while fathers oppose FGM, family females have the ultimate say: “*FGM is entirely decided upon by our mothers; our fathers have no involvement in this decision, even though they don’t agree.*” (A participant from Group 1).

### Category 3: experience of FGM among high school girls

Participants were asked to share their personal experiences with the effects of circumcision. However, while discussing they didn’t tend to specify if the symptoms are personal experiences, the experiences of their family members and relatives have dominated their conversations. The results were summarized in the following sub-categories: the negative effects of FGM, and the perceived positives of FGM.

### The negative effects of FGM

The majority of participants noted that circumcision causes unfavorable side effects. Both early and late negative effects have been listed. One participant explained the consequent feelings most girls go through: “*The girl feels happy in the beginning because of the henna and gifts, but she becomes afraid when the midwife comes with the blades and razors, and later on after the procedure she will feel pain, suffer from urination, and have walking difficulties*” (a participant from group 1). With the rest, proceed on mentioning many immediate and late-onset side effects. Labor difficulties are the most noticeable late adverse effect that was noted.

“*Tetanus is a side effect of circumcision, which can lead to death*” (A participant from Group 3).

“*Circumcision can affect the first night of marriage*”. (A participant from group 4)

”*Those side effects will decrease with time*” (A participant interrupted trying to defend their norms.)

Similar to the second group, which identified fear as a side effect, the majority of the girls in the fourth group specified mental health issues.

“*Some girls develop depression*” (A participant from group 5).

Some of them asserted that circumcision could be redone if done incorrectly initially or if the freshly circumcised girl experienced an accident. A different girl in the same group, though, claimed that “*circumcision is a religious practice, so it has no negative side effects*”. Another participant from a different group also denied the side effects, yet for a different reason; “*there are no obvious side effects that can be mentioned because circumcision is done when the girl is young and not aware*” (A participant from Group 3).

### Perceived positives of FGM

Although the majority of the participants didn’t address any advantages to the FGM, still a few of them perceived FGM more positively. One of the participants from group three who said she was unsure of the benefits of circumcision declared, “*I will continue doing it*”. A member of group IV who stated that it is not advantageous added “*It is also not harmful*”.

A different participant from group four who consented to keep being circumcised noted that “*circumcision delays the onset of labor, so the women don’t give birth alone*”.

### Category 4: attitude towards FGM

The study participants were then stimulated throughout the discussion to share their beliefs and attitudes toward uncircumcised women and towards FGM itself. Two sub-categories emerged from this theme; discrimination or difference between circumcised and uncircumcised girls and the need to stop FGM.

### Differences between circumcised and uncircumcised women

Upon directly asking the participants if they think there is a difference between the circumcised and the uncircumcised, the majority stated that there is no difference. Nonetheless, when they got involved in the discussion, they disclosed many contradictory beliefs. In furtherance, a number of them connected this practice to religion since they claimed it was required religiously. They noted that the majority of men choose to marry circumcised women, and some of the participants described some effects of circumcision on the body’s physical and behavioral characteristics.

“*The uncircumcised girls are more obese than the circumcised ones*.” (Three participants from group 4).

“*The circumcision makes the girl quieter*”. (A participant from Group 5)

”*I noticed the uncircumcised girls protrude their tongues*”. (A participant from group 4)

”*The baby of the uncircumcised girl will die*”. (A participant from Group 1)

”*The pharaonicly circumcised girls have one opening for peeing and periods, which are left separate in the uncircumcised girls*” (A participant from Group 4).

Moreover, uncircumcised girls were regarded as uncivilized by part of the community and were commonly abused verbally and disparaged.

“*The community here believes that the uncircumcised girls are disrespectful*” (A participant from group 1).

### The need to stop FGM

The majority of participants were in agreement that female circumcision should end. She pointed out that she will never let her daughters go through this bad experience she was forced to.

“*No, I will not circumcise my daughter; this is a bad thing, and she shouldn’t go through with it*.” (A participant from group 5).

“*Circumcision should stop because it’s difficult on the girls*.” (A participant from group 1).

Contrary to what is believed among their colleges, three participants from Group five indicate that they will avoid circumcising their daughters based on religious beliefs that prohibit it. Another three participants from Group 1 said that they don’t prefer circumcision, but they will do it if their grandmothers forced them.

“*I will circumcise my daughters if my grandmother forces me to*.“.

Only one girl replied, “*I will circumcise my daughters anyway, because it doesn’t matter*.”

## Discussion

The aim of this study was to assess high-school girls’ perspectives, knowledge, and experience of female genital mutilation. We explored as well the level of education of the parents in order to find an association between literacy and this illegal practice; however, being the daughter of educated parents or not did not change the destiny of the female of being circumcised, as all of our participants were high school girls who found to be undergone FGM (100%). This prevalence is considerably high when compared to other Arabic nations; comparable research found that FGM prevalence among girls aged 15 to 45 was reported to be 8% in Iraq and 19% in Yemen [17].

Our study revealed that most of the females first became acquainted with FGM from their mothers and grandmothers as a praised social habit. Nevertheless, one of the participants who was strongly opposing FGM mentioned that she originally heard of it from social media as a forbidden thing, and this emphasizes the importance of early enlightenment of the dangers of such bad practices at a young age.

Speaking about the general understanding of the nature of the procedure, the results showed that girls suffer from an obvious confusion about what is being cut out and what is certainly performed, even though all of the participants were circumcised. Lack of women’s knowledge about the types of FGM had been reported before in a study in eastern Sudan [19]. Other studies in Nigeria, Iraq, and Egypt concluded this same finding [17, 20, 21] of being subjected to an excision and not being fully aware of what is exactly cut out—not even after puberty—gives us a clue of how this practice violates the autonomy and dignity of females.

Mentioning the autonomy of females, here we come to a crucial concept regarding FGM decision makers: those who we can impact the most in order to stop the abuse. All of the participants agreed that mothers and grandmothers are the decision-makers, and they definitely stand against any stopping trials from the fathers or whoever incriminates the practice. We find this particular finding to be interesting, and it was also noticed in a study in Kurdistan region [17]. Some studies emphasize the male role in decision-making [22], suggesting internalized oppression as the concept behind this unbroken chain of women abusing women. According to them, women were misinformed, believed throughout history, and then stereotyped negative thoughts about themselves being uncircumcised [17, 23].

Gladly, there is an agreement among participants that doctors are not included in this procedure. Moreover, some girls mentioned that this referred to the illegality of FGM. Traditional circumcisers are actually the key players in the majority of nations where FGM is practiced [20, 24] which jeopardizes the safety and hygienicity of the procedure. Notably, physicians in their clinics are predominantly responsible for performing FGM in a few countries, like Egypt [21].

In terms of sociocultural aspects, there was a spectrum of opinions regarding the religious background of female genital cutting, ranging from thinking that FGM is a part of the religion to thinking that FGM is prohibited in religion; a previous Sudanese study reported religion frequency as a cause for FGM to be 26% [19]. This finding is particularly common in Iraq (2.8%) [24] and Nigeria (10%) [25]. Islamic religious leaders have various opinions regarding FGM, with some criminalizing it and others encouraging it as a religious practice [26, 27].

Moreover, stigmatizing uncircumcised women as being unclean and lowering their chances in marriage is a repeatedly reported social aspect [28]. However, there were also some comments by the participants about the physical appearance and attitudes of the uncircumcised girls, such as being overweight, having a tendency to protrude their tongues, and having bizarre social interactions. These results are unique to this study and need further investigation in other regions of Sudan.

Despite the fact that not all of the participants have showed complete disapproval of FGM as a practice, there was a general consensus among the girls regarding its bad physical and psychological outcomes, including both the early and the late side effects. After investigating the nature of these side effects, tetanus, urination difficulties, depression, difficulties in intercourse, and difficulties in labor were all mentioned as possible side effects. As premarital sex is prohibited in such communities, the young participants’ thoughts about the side effects were obtained from education rather than their personal experiences with sexual life and birth giving. However, despite a bit of reserve would be an obstacle, further research about the sexual life and obstetric health of these women need to be conducted. This profile of complications is universally reported in almost all FGM reports [3, 7, 29]. Notably, participants in the study tended to mention the side effects as a general phenomenon while tagging themselves as a third party, without indulging into their own experiences. Therefore, we could only report their reflections without the ability to segregate their own experiences from those they have noticed.

None of the participants mentioned the immediate side effects like fever, prolonged bleeding, or vulval swelling, which are similar to the results of a study in Iraqi Kurdistan Region [17]. In our opinion, the relatively good knowledge of side effects compared to the widespread practice emphasizes the importance of reforming culture through law rather than only raising awareness, as it could not solely reduce the habit. This opinion is supported by the clear decrease in the prevalence of FGM in Egypt after its criminalization in 2008 [30]. Interestingly, reducing sexual desire which was mentioned by other studies as a common side effect to FGM [17], appears to be an important drive for continuing the practice in this village. They believe that uncircumcised women have an exaggerated sexual desire that could bring shame and disgrace to the family if left unrestrained.

In concluding our interviews, we investigated the future vision of the participants regarding their daughters’ destiny with FGM. Unfortunately, almost half of our participants are planning to perform FGM on their daughters, and the other half have shown a hesitant rejection of it with the ability to change their mind under family pressure.

Unlike another study that was carried out in the UAE and was matching a line of other previous studies, where the trend of FGM is thought to be on the way to being eradicated [17, 31], here however, we find that the change is yet too early to start. However, we recommend that the change start with the elderly decision makers, namely mothers and grandmothers; as the FGM is usually operated in a young age, and fathers’ thoughts have not been found a major contributor. Although raising awareness through education, community workshops and dialogues would be helpful, we think that this issue is very culturally complicated and deep that education alone could take several decades to make a considerable change; based on this finding, we recommend advocating strict legal measures with the support of the community leaders and this could make a dramatic change in the prevalence of this harmful practice. Finally, as most of the communities that embrace FGM are practicing under the cover of religion and tradition, collaborating with religious and traditional leaders in the process of eliminating it would shorten the path of change.

### Limitations

An in-depth assessment of the viewpoints of girls who have undergone FGM was made possible with the aid of a qualitative study design. Also, using focus groups and including individuals who were of a similar age promoted more involved discussions and looked at different aspects of FGM issues. However, this study has had some limitaions. First of all, the study focused on a specific age category of young non-sexually active girls, which unpermitted us to hear from older women who has personally faced the actual and major physical and psychological impact of FGM regarding sexual life and birth giving experience. Secondly, despite that the participants were generally welcoming, they were a little bit reserved and shy about the sensitive questions describing sexual organs and sex life. Furthermore, response bias may be anticipated due to two possible limitations. The first one is that, despite the moderators being students which would probably put the participants in some ease, they were still considered medical experts who are opposing the practice. The other possible limitation is that participants who are aware of legislation making the practice of FGM illegal may be reluctant to declare their support for the procedure; these two possible limitations were overcome by the open discussion and the introductory explanation that made the participants aware of the research goals and ethics, which comforted them to open up safely. In addition, this scientific study has been conducted in only one village, despite the cultural similarity predicted from all the rural areas in Sudan.

## Conclusion

According to the study’s findings, FGM is mostly a decision made by mothers and grandmothers as a traditional practice, with males generally disapproving it. The exact executors are unregistered midwives; high school girls appear to be aware of the doctor’s involvement, with some suggesting medicalization of FGM. The study reported that early side effects like pain, difficult urination, and walking are common and frequent, and there was knowledge about late ones. The participant’s knowledge of the dangers of FGM was better than expected given the high prevalence. The participants had an optimistic outlook on future efforts to end the practice. Nonetheless, the majority of them confessed and explained that they would continue if pressured by their grandmothers. All high school girls who participated in this study were well informed about FGM, and they communicated the experiences they lived with or noticed openly.

## Data Availability

The datasets generated during this study are available upon request from the corresponding author.

## Abbreviations

FGM: Female Genital Mutilation
WHO: World Health Organization
DFID: The Department for International Development
USAID: US Agency for International Development
FGDs: Focus group discussion
